# Metamaterial-based real-time communication with high information density by multipath twisting of acoustic wave

**DOI:** 10.1038/s41467-022-32778-z

**Published:** 2022-09-02

**Authors:** Kai Wu, Jing-Jing Liu, Yu-jiang Ding, Wei Wang, Bin Liang, Jian-Chun Cheng

**Affiliations:** Key Laboratory of Modern Acoustics, MOE, Institute of Acoustics, Department of Physics, Collaborative Innovation Center of Advanced Microstructures, Nanjing University, Nanjing, 210093 P. R. China

**Keywords:** Acoustics, Electronic devices, Metamaterials

## Abstract

Speeding up the transmission of information carried by waves is of fundamental interest for wave physics, with pivotal significance for underwater communications. To overcome the current limitations in information transfer capacity, here we propose and experimentally validate a mechanism using multipath sound twisting to realize real-time high-capacity communication free of signal-processing or sensor-scanning. The undesired channel crosstalk, conventionally reduced via time-consuming postprocessing, is virtually suppressed by using a metamaterial layer as purely-passive demultiplexer with high spatial selectivity. Furthermore, the compactness of system ensures high information density crucial for acoustics-based applications. A distinct example of complicated image transmission is experimentally demonstrated, showing as many independent channels as the path number multiplied by vortex mode number and an extremely-low bit error rate nearly 1/10 of the forward error correction limit. Our strategy opens an avenue to metamaterial-based high-capacity communication paradigm compatible with the conventional multiplexing mechanisms, with far-reaching impact on acoustics and other domains.

## Abstract (web summary)

Here, the authors demonstrate multipath twisting of acoustic waves with a thin metamaterial layer enabling high-speed transfer of information with no time-consuming post-processing or sensor scanning, showing important application potential in underwater communication.

## Introduction

It is a fundamental problem in wave physics to continuously boost the transmission capacity and speed of the information carried by waves, which are highly desired for a plethora of applications. A representative example is the underwater communication dominated by acoustic wave due to the low absorption and scattering effects as compared to its electromagnetic counterparts^1–5^. Given the inherently low frequency and speed of acoustic waves and their lack of polarization, however, breaking through the current information transmission efficiency has been a long-standing challenge crucial for acoustic communications. Recently orbital angular momentum^6–9^ (OAM)-based acoustic communication is proposed and implemented which uses space as a new dimension independent of phase and amplitude for encoding information. For vortex beam imprinted with specific OAM, its typical spiraling phase can be mathematically expressed as $\exp ({{{\rm{i}}}}l\theta)$, where *l* is the topological charge and *θ* is the azimuthal angle. Based on the orthogonality of OAM, different modes of OAM beams carrying independent information can be multiplexed into a single path without inter-modal crosstalk. This enables significant boost of the acoustic communication capacity but needs postprocessing of signals received with complicated sensor arrays^10^ or multi-layered metamaterials with de-multiplexing capability limited by insertion loss and diffraction effect^11^.

More importantly, the OAM multiplexing^12–15^ strategies rely on multiple coaxially-overlapped twisted beams used as orthogonal communication channels, while leaving the potential of non-coaxial beams to further boost acoustic communication efficiency unexplored. In such cases, the channel number has to be increased by including more high-order OAM modes, leading to more severe diffraction and spatial aliasing effect, which limit the maximum number of available communication channels^16–18^. Although the possibility of information multiplexing/demultiplexing based on non-coaxial OAM beams has been proven in optics^19–22^, it is not feasible to directly translate the same mechanism to acoustics considering the fact that complicated equalization algorithm^23–25^ based on time-consuming computation is needed for counteracting the diffraction-induced crosstalk effect which is much stronger for acoustic waves. On the other hand, the passive devices previously proposed for decoding optical OAM beams in real-time are too bulky to straightforwardly apply to acoustic waves with macroscopic wavelength^26–31^. These pose fundamental limits on the further boosting of the spatial information density (defined as the number of available channels per unit transmitter/receiver area) one can achieve with the existing mechanisms of acoustic communications.

In this article, we theoretically propose and experimentally demonstrate a mechanism that uses multi-path twisting of acoustic waves for realizing real-time free-space communication in a passive postprocessing-free and sensor-scanning-free paradigm. By combining the advantages of both coaxial and non-coaxial vortex beam multiplexing, we break through the limits of information transfer capacity in the current spatial multiplexing-based mechanisms. Besides, the introduction of non-coaxial acoustic vortex beam as a new encoding degree of freedom also helps to reduce the dependence on high-order OAM beam and significantly increase the effective communication distance and signal-to-noise rate (SNR). A monolayer metamaterial-based demultiplexer is designed to decode the information carried by such synthesized beams in real-time despite its compact size in all three dimensions, resulting in unprecedentedly high information density in space. In particular, such metamaterial-enabled^32,33^ mechanism is innately robust to the multi-path effect that would otherwise be significant for a small-scaled system and notably lower the communication accuracy by weakening the contrast between different paths in conventional designs. Furthermore, our strategy is still compatible with the traditional equalization mechanism used in conventional multiple-input multiple-output (MIMO) spatial multiplexing techniques, offering the possibility to further enhance the communication efficiency. We demonstrate the performance of a high spatial information density communication system designed based on our proposed mechanism both numerically and experimentally via distinct example of a complicated image transmission, which shows the high accuracy and high efficiency of our proposed mechanism.

## Results

### The theory of multipath twisting of acoustic wave

**Fig. 1:**
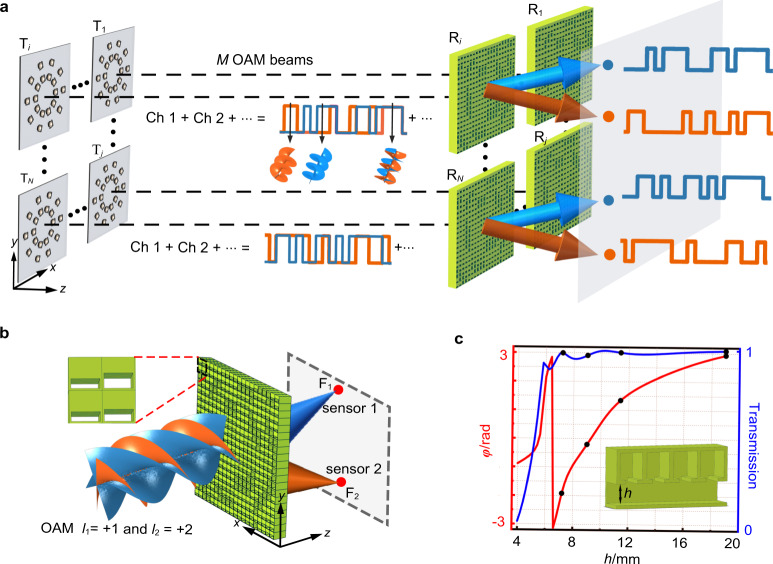
Schematic of free-space acoustic communication with high spatial information density by multi-path twisting of acoustic wave. **a** The designed communication system consists of *N* pairs of transmitting and receiving units with each transmitting unit containing *M* coaxial vortex beams with different orders, resulting in a total number of *NM* independent data channels. At the receiving terminal, real-time and passive decoding of the transmitted information is realized by using *N* metamaterial-based demultiplexers with planar profile and ultrathin thickness. Ch: channel. **b** The schematic of simultaneous demultiplexing of synthesized vortex beams and beam focusing into prescribed locations with single metamaterial-based demultiplexer. Inset: partially enlarged view of the metamaterial layer. **c** Simulated phase shift and transmission provided by the metamaterial unit cells with different structural parameter *h*. Inset: section view of an individual element.

Our proposed mechanism for establishing a real-time acoustic communication with high spatial information density is schematically illustrated in Fig. 1a. In our multi-path system, *N* transmission paths are established between *N* pairs of transmitting and receiving units, referred to as T_*i*_ and R_*i*_ respectively with $i\,=\,1,\,2,\,{{{\ldots }}},\,N$. Each transmitting unit emits a synthesized acoustic beam formed by coaxially overlapping vortices with different orders *l*_*m*_ with $m\,=\,1,\,2,\,{{{\ldots }}},\,M$, which are used as independent channels for encoding different information. As a consequence of combining coaxial and non-coaxial vortex beams, the total number of communication channels reaches ${{{{NM}}}}$, which is a remarkable boost in comparison to the existing acoustic communication mechanisms^10,11,34^ such as those using OAM-multiplexing/de-multiplexing. Although OAM states reside in an infinite dimensional Hilbert space, the production and recognition of vortex beams with high order, which needs to be ${{{{NM}}}}$ for achieving equivalent information capacity, is challenging due to the strong diffraction effect^17^. It should be noted that, however, the diffraction effect of vortex beam is also unavoidable in our mechanism, which leads to spreading out of beam during propagation and causes the receiving unit R_*i*_ to also pick up the information carried by encoded vortices emitted from transmitting units other than T_*i*_. This is responsible for the channel crosstalk (or interference) effect that degrades the received signals.

Despite the seeming similarity, it is worth stressing that our proposed mechanism differs intrinsically from the existing spatial multiplexing-based mechanisms of information transfer. One of the most important features of our system is the record-breaking high spatial information density which is ensured by more available channels and the compactness of both the transmitting and receiving terminals. Physically, the shrinking of the transverse size of emitting source and the increase in vortex order dramatically increase the diffraction of information-carrying beams and subsequent crosstalk effect, which conventionally needs to be counteracted via complicated postprocessing of the signal measured by large-scaled sensor array and hinders downscaling of the transmitting/receiving units. In contrast to the existing optical communication systems with bulky size, our mechanism uses a rationally designed metamaterial with compact size in all three dimensions to receive and decode the information carried by the transmitting acoustic beam. Such metamaterial-enabled de-multiplexing mechanism can be simply understood as follows. The whole metamaterial layer comprises *N* subunits, each of which is judiciously designed to redistribute the energy of a synthesized vortex beam normally impinging on it such that the *l*_*m*_*-*th OAM beam is effectively untwisted and converged into a prescribed focal point (marked by F_*m*_) at an output plane, as illustrated in Fig. 1b. It can therefore be expected that the acoustic signal received by a single sensor placed at F_*m*_ has the original encoded information and an amplitude augmented by a constant factor, which simultaneously enhances the SNR and maintains the compatibility with the conventional multiplexing techniques using phase, amplitude, frequency, etc^35–37^.

More significantly, the designed metamaterial has strong spatial selection since any oblique incidence of vortex beam will not notably affect the acoustic intensity at the prescribed focal points. With innate robustness to the growth in undesired crosstalk effect, our mechanism allows design of a simple and low-cost passive system for real-time communication of information with high spatial density in a sensor scanning-free and postprocessing-free paradigm. It is also noteworthy that our proposed mechanism is inherently compatible with the conventional MIMO signal processing to further equalize the crosstalk between different channels and substantially reduce the computational complexity by simplifying the channel matrix as ensured by the orthogonality among the OAM beams with *M* different modes (see Supplementary Note 1 for more details). Furthermore, thanks to the monolayer metamaterial design with high transmission and parallel interaction between all its unit cells and the incident acoustic beam, the proposed system can be readily extended to have larger number of communication channels while reducing the increased hardware complexity and energy consumption in conventional communication mechanisms.

In our designed acoustic communication system, the information-carrying acoustic beam emitted from the transmitting unit T_*i*_ is generated by using an active transducer array with each transducer being individually controlled by a single-chip microcomputer Arduino Mega 2560, which independently modulates the amplitude and phase of the signal (see Supplementary Note 2 for more details), as shown in Fig. 1a. Then the acoustic pressure distribution on an individual transmitting unit is $p(\theta,t)=\mathop{\sum }\nolimits_{m=1}^{M}{A}_{m}(t){e}^{{{{{{\rm{i}}}}}}({l}_{m}\theta+{\phi }_{m}(t))}$, which produces a multiplexed acoustic vortex beam that will be subject to diffraction effect when propagating in free space. Here the amplitude *A*_*m*_(*t*) and phase *ϕ*_*m*_(*t*) are time-dependent and can be modulated by existing modulation techniques such as phase-shift keying (PSK), amplitude-shift keying (ASK) and quadrature amplitude modulation (QAM) for further improving the data capacity and spectral efficiency. In the current study, we choose to use binary amplitude-shift keying (2ASK) modulation technology to encode information into the amplitude of the produced acoustic vortex beams as different sequences of “0” and “1”. At the receiving end, the transfer function of the single metamaterial-based demultiplexer can be expressed as

1 $${\Psi }=\exp ({{{{{\rm{i}}}}}}\varphi )=\mathop{\sum }\limits_{m=1}^{M}\exp ({{{{{\rm{i}}}}}}k{r}_{m}-{{{{{\rm{i}}}}}}{l}_{m}\theta )$$

where *k* is the wavevector in free space and ${r}_{m}=\sqrt{{(x-{x}_{m})}^{2}+{(y-{y}_{m})}^{2}+{(z-{z}_{m})}^{2}}$ is the distance between the demultiplexer and the focal point (*x*_*m*_, *y*_*m*_, *z*_*m*_) of the *l*_*m*_-th OAM (detailed design of demultiplexer can be seen in Supplementary Note 3). This transfer function simultaneously removes the OAMs of all the vortex beams and converges the energy of different OAM modes into their respective detection points^38^. Note that our mechanism eliminates the coupling among demultiplexed beams and thus allows freely manipulating the propagation path of every output beam regardless of the topological charge. Based on this, we further extend the output region to the whole three-dimensional (3D) space behind the demultiplexer, which boosts the channel capacity as well as lowers the crosstalk effect. As a practical implementation, each metamaterial unit cell of the demultiplexer is designed as a hybrid structure composed of a side-loaded resonating cavity array and a straight pipe for modulating the propagation phase of transmitted wave and enhancing the transmission efficiency respectively. We choose to modulate the height *h* of straight pipes to effectively realize the manipulation. The resulting metamaterial unit cell can thus be modified to produce arbitrary phase shift within the full 0-to-$2\pi$ range while keeping a near-unity transmission as required by our mechanism (see Fig. 1c). Instead of continuously varying the structural parameters of each unit cell, in our experiments we only use four kinds of hybrid structures as basic building blocks (marked by four dots in Fig. 1c), which substantially simplifies the design and reduces the possible experimental errors (see Supplementary Note 4 for details). Based on this design, the metamaterial can precisely decode the data carried by synthesized vortex beam and the time-domain signals in *NM* channels can be received by ${{{{NM}}}}$ microphones located at focal points, as will be verified via numerical simulations and experimental measurements in what follows.

### Experimental realization of real-time communication using multipath multiplexed vortex beams

**Fig. 2:**
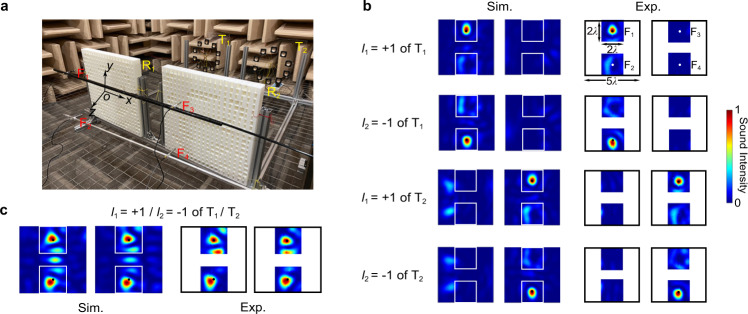
Experimental setup for our proposed communication system utilizing multipath twisting of acoustic wave and typical measured results. **a** Photograph of the experimental setup of 2-paths 4-channels communication system. Two metamaterial-based demultiplexers are placed in alignment with corresponding transmitters with a relative distance of 1.2 m. Four microphones are placed at four predesigned focal points marked by F_1_–F_4_ to receive the modulated signals. **b**, **c** The comparison between the normalized simulated and measured sound intensity distributions of four 20 × 20 cm^2^ square regions centered at four focal points when turning on **b** a single channel from T_1_ or T_2_ with OAM mode *l*_1_ = +1 or *l*_2_ = −1 and **c** all four channels in two paths simultaneously.

The experimental setup for demonstrating the effectiveness of our proposed mechanism of free-space communication with multipath twisting of acoustic wave is depicted in Fig. 2a. The measurement is carried out in an anechoic chamber for eliminating the undesired reflections from the boundaries. For simplicity while without losing generality, in the current study we choose to build a communication system with four independent channels, which includes two pairs of transmitting and receiving units with each emitting two co-axially overlapped OAM beams with *l*_1_ = +1 and *l*_2_ = −1 (viz., *N* = *M* = 2). In our simulations and experiments, the working frequency *f*_0_ is set to be 3430 Hz (corresponding to a wavelength *λ*_0_ of 10 cm in air). The distance between the centers of T_1_ and T_2_ is 0.7 m. The multiplexed vortex beams emitted from T_1_ and T_2_ with radial sizes of 0.15 m are propagating toward the receiving units R_1_ and R_2_ over a transmission distance of 1.2 m (12*λ*_0_), and the spreading and overlapping of vortex beams persist during propagation which may result in channel crosstalk such as in conventional MIMO communication (see Supplementary Note 5). However, our mechanism with spatial selectivity offers the robustness to diffraction-effect-induced crosstalk and ensures the accuracy of communication, as will be discussed later. At the receiving end, two demultiplexers are placed in alignment as a single-layered metasurface to detect and decode the data of four channels. For each OAM mode, we choose a specific focal point located in the 3D space behind the metamaterial layer, i.e., point F_1_ (0, 0.15 m, 0.2 m) for OAM mode *l*_1_ = +1 from T_1_, F_2_ (0, −0.15 m, 0.2 m) for OAM mode *l*_2_ = −1 from T_1_, F_3_ (0.7 m, 0.15 m, 0.2 m) for OAM mode *l*_1_ = +1 from T_2_ and F_4_ (0.7 m, −0.15 m, 0.2 m) for *l*_2_ = −1 from T_2_. After interacting with the compact metamaterial with each subunit of thickness and width of *λ*_0_/2 and 5*λ*_0_ respectively (i.e., 20 × 20 hybrid structure unit cells), the two-path multiplexed vortex beams are demultiplexed and converged into four focal points and finally received by four microphones. Such a device with transverse size of 5*λ*_0_ × 5*λ*_0_ can realize effective demultiplexing of the multiplexed vortex beams after long-distance propagation while ensuring high spatial information density. Without complicated and time-consuming post-processing algorithm or sensor scanning, our methodology enables direct information decoding by using a single sensor to pick up the sound signal in the focus. Besides, thanks to the remarkable enhancement of local acoustic intensity, the SNR of receiving signal increases dramatically, ensuring longer effective transmission distance and lower bit error rate (BER) in acoustic communication process.

In experiment, the spatial distribution of sound intensity on the output plane after the metamaterial layer is measured by using a 1/4-in. free-field microphone (Brüel & Kæjr type-4961) which is attached to a 3D stepping motor to scan the target regions point by point. Figure 2b shows the comparison between the simulated and measured spatial distributions of acoustic intensity of four 20 × 20 cm^2^ square regions centered at four focal points on the output plane when only a single communication channel is “turned on”, e.g., in the upper figure we only transmit acoustic signal encoded into a vortex beam with *l*_1_ = +1 from T_1_. Next, we also measured the sound intensity profile of the output plane when all the four OAM channels are turned on simultaneously and plotted the typical results in Fig. 2c. By separately inspecting the output wave field produced by each vortex beam, we prove both numerically and experimentally that the designed metamaterial focuses the acoustic energy into the target focal point with high efficiency and precision regardless of the number of working channels. Notice that the practical focal positions in the detection plane slightly offset especially for multi-mode OAM demultiplexing, which is owing to the superposition of target mode and additional OAM mode generated by the demultiplexer. However, due to the null center of OAM mode the sound signals at the foci received by the fixed single microphone will not be affected by the additional OAM mode despite the foci offset. The high spatial selectivity of metamaterial on the incidence direction of vortex beam is verified by the fact that the acoustic intensities at the rest focal points remain negligible as expected. This accounts for the strong robustness of our mechanism against the crosstalk effect caused by inter-channel interference, as will be demonstrated in what follows.

To quantitatively evaluate the strength of inter-channel crosstalk in our designed system, we experimentally measured the power leakage between the four communication channels and calculate the crosstalk of each channel as listed in Table 1. The crosstalk for a specific OAM channel *l*_*m*_ can be calculated by ${P}_{l\ne {l}_{m}}/{P}_{l={l}_{m}}$, where ${P}_{l\ne {l}_{m}}$ is the sum of the received power of channel *l*_*m*_ when all channels except for channel *l*_*m*_ are opened separately, and ${P}_{l={l}_{m}}$ is the received power of channel *l*_*m*_ when only channel *l*_*m*_ is turned on. We observe that the crosstalk of four OAM channels is below −10 dB, which ensures the high accuracy and robustness of our communication system. Notice that the crosstalk effect caused by the diffraction effect is not trivial in the system shown in Fig. 2a, which would otherwise degrade the received signal in the absence of our proposed de-multiplexing mechanism. On the other hand, the crosstalk effect is not perfectly uniform for all the communication channels as in an ideal model, which may result from the experimental error such as imperfect sample fabrication and alignment.

**Table 1:** Power transfer and total crosstalk of each OAM channel

Power Transfer (dB)	T_1_ *l*_1_ = +1	T_1_ *l*_2_ = −1	T_2_ *l*_1_ = +1	T_2_ *l*_2_ = −1	Crosstalk (dB)
R_1_ *l*_1_ = +1	−7.59	−29.62	−21.04	−29.07	−12.33
R_1_ *l*_2_ = −1	−21.39	−9.64	−27.45	−33.46	−10.57
R_2_ *l*_1_ = +1	−24.11	−33.72	−8.20	−29.58	−14.47
R_2_ *l*_2_ = −1	−32.44	−31.54	−20.37	−8.5	−11.31

**Fig. 3:**
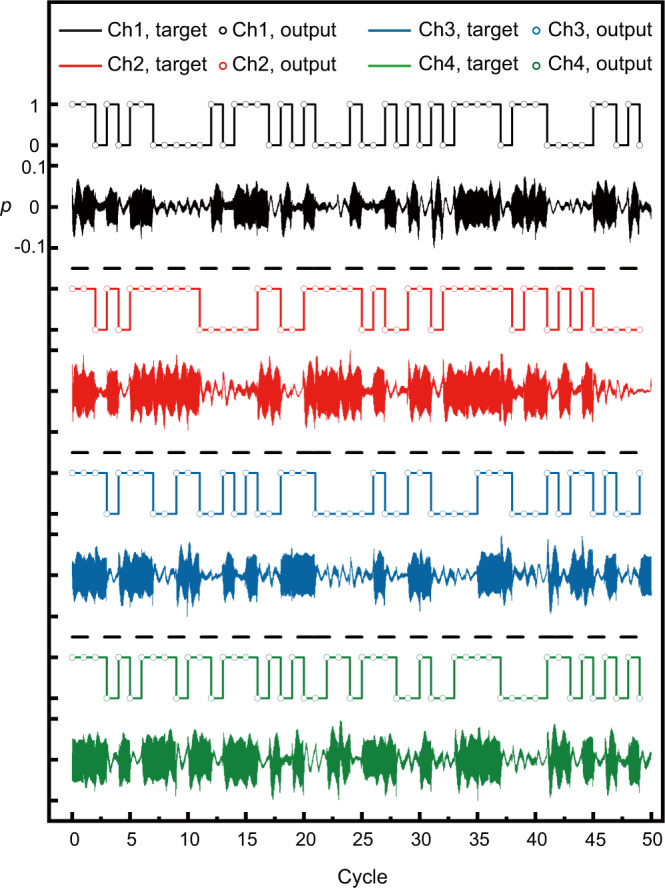
Real-time communication of four 50-bit data streams in parallel. The comparison between the target data stream and the received output in the four channels, and the measured time-domain signals in each pulse period in four channels. Each of two transmitting units emits a synthesized vortex beam with *l*_1_ = +1 and *l*_2_ = −1, opening four independent channels marked by Ch1, Ch2, Ch3, and Ch4 respectively.

We first demonstrate the effectiveness of our proposed mechanism via a simple example of independent transmission of four 50-bit data streams in parallel over a communication distance of 12*λ*_0_, as illustrated in Fig. 3. In our implementation, four independent channels in two paths, marked as Ch1 to Ch4, are utilized to transmit two-path synthesized vortex beams, with which we could encode four data streams into the amplitude of each vortex beam in 2ASK format (see Supplementary Note 6 for details). The data-carrying signal is in pulse modulation with a pulse period of 20*T*_0_ (*T*_0_ refers to the period of carrier wave) and each pulse cycle contains 1-bit data such that the communication speed of our system is 686 bit/s. The time-domain signals received at four focal points are also plotted in Fig. 3, corresponding to the four data streams respectively. For the purpose of extracting data streams from the time-domain signals, we perform the cross-correlation by multiplying the received signals with an ideal sinusoidal signal of identical frequency (see Supplementary Note 7 for details). The decoded data streams in four channels are plotted in Fig. 3 (marked by dots) in comparison with the target signal. Obviously, the decoded information streams are consistent well with the input ones without any distortion, indicating that our proposed mechanism allows to achieve real-time and high-precision decoding of information transmitted over four independent channels even in the absence of complicated postprocessing indispensable for conventional mechanisms.

Notice also that the number of channels, working frequency, and digital modulation techniques in our scheme, are chosen for facilitating the proof-of-concept experiment rather than attaining an optimal performance, thereby leading to the relatively limited communication speed. Thanks to its compatibility with existing communication techniques, our mechanism enables significantly boosting the communication capacity by *NM*-fold. This suggests that by adjusting the working frequency and digital modulation techniques, the communication speed of our design can be easily improved to outperform the conventional acoustic spatial multiplexing communication systems (see Supplementary Note 8 for design example of realizing higher communication speed). Therefore, our mechanism opens an avenue to high-capacity communication paradigm, where the high capacity is realized by a larger number of achievable OAM channels and the compatibility of our proposed mechanism with existing modulation techniques.

**Fig. 4:**
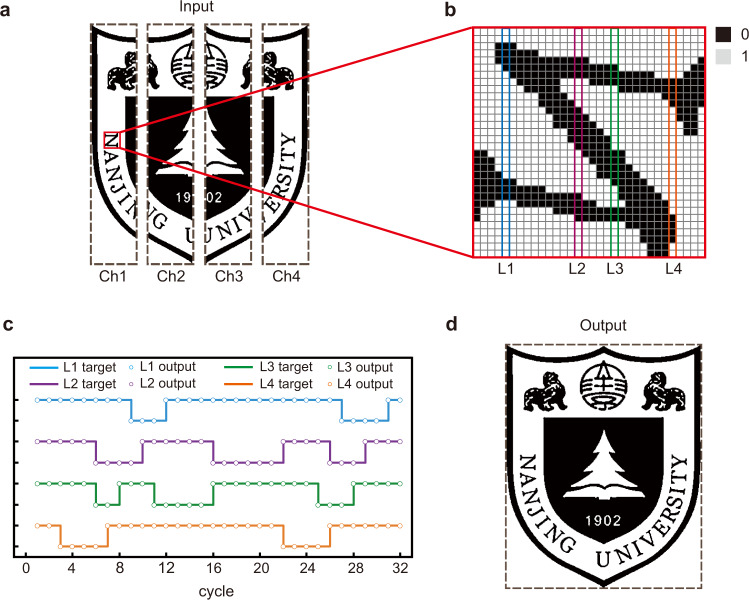
Experimental results of image transmission of a binary image of Nanjing University logo. **a** The original input image which is divided into four separate parts for a parallel transmission along four independent channels (viz., Ch1, Ch2, Ch3, and Ch4 respectively). **b** The enlarged view of square region marked by red line in **a**. The two colors of pixel are encoded by binary information “0” and “1”. **c** The target and output data streams of four lines in **b**, marked by L1, L2, L3, and L4. **d** The output image reconstructed from four data streams received in real-time by four sensors located at focal points. Permissions for use of the Nanjing University logo in this figure were obtained from Nanjing University. All rights reserved.

Next, we experimentally showcase a more practical example of real-time transmission of a complicated binary image including 462 × 368 pixels. As shown in Fig. 4a, the target image of a school badge is divided into four parts and transmitted through four OAM channels simultaneously. Therefore, each channel will transmit 42504-bit 2ASK data stream. Figure 4b shows the enlarged view of a specific square region marked by the red square in Fig. 4a, where the binary information “0” and “1” represent two different colors of each pixel. The decoded information of four lines L1, L2, L3, and L4 extracted from the time-domain signals is depicted in Fig. 4c, where the target data stream is also presented for comparison. By assembling four measured data streams together, we obtain the output image and plot the results in Fig. 4d, which exhibits the high quality of image transmission. Thus, by utilizing two-path twisting of acoustic wave we realize the real-time transfer of school badge image with high spatial information density. The real-time communication is evidenced by the fact that there is no time delay caused by time-consuming computer-aided postprocessing as in the conventional active mechanisms, except for the time of wave propagation between the transmitting and receiving terminals.

**Fig. 5:**
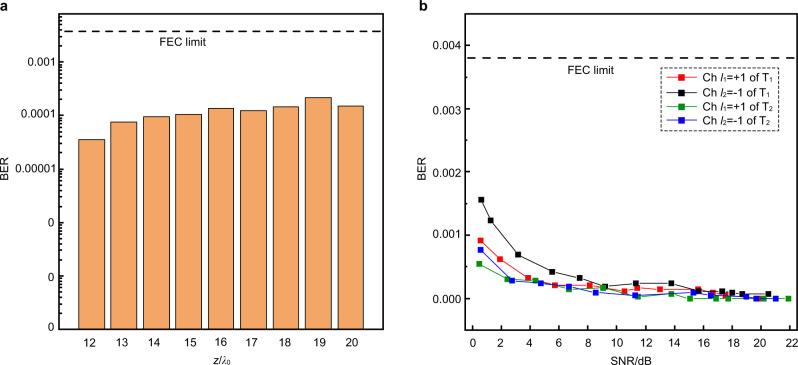
The measured BERs for the transmission of the binary image as a function of the transmission distance and SNR respectively. **a** Measured BERs of school badge image versus transmission distance. **b** The measured BERs curves of four channels versus SNR.

In order to give a quantitative estimation of the accuracy of data transfer in our proposed mechanism under longer transmission distances, we increase the transmission distances from 12*λ*_0_ to 20*λ*_0_ and employ BER to characterize the performance of our communication system. Here, the BER is defined as the ratio of the number of error symbols to the total number of all transmitted symbols. Figure 5a shows the measured BERs for the transmission of the binary school badge image as a function of the transmission distance. It is apparent that the measured BER curve is much lower than the forward error correction (FEC) limit^39^ of 3.8 × 10^−3^. Interestingly, when the transmission distance of our communication system reaches 20*λ*_0_ the mainlobe of vortex beam has already exceeded the receiving region and non-coaxial vortex beams have overlapped severely in space. However, the demultiplexer can still work and keep superior performance, which suggests our metasurface-enabled demultiplexing mechanism has inherent robustness to channel crosstalk. To further characterize the performance of our communication system, we vary the SNR of the received signal by increasing the ambient noises and experimentally measure the BERs of recovered binary image versus SNR under a transmission distance of 12*λ*_0_. From the typical results in Fig. 5b, we can clearly observe that the BERs of four channels increase with the decrease of SNR, as expected. Nevertheless, the BER of the whole communication system is still much lower than the FEC limit even at extremely low SNR level, which demonstrates the important antijamming capability of our proposed system. Although here we only demonstrate a communication system with a relatively short transmission distance of 20-wavelength in experiment for facilitating the experimental fabrication and measurements, our mechanism can apply to realize accuracy and remote communication over a km-scale distance with optimal transmitting/receiving array dimensions (see Supplementary Note 9). Due to the fact that the OAM beams with small topological chargers are less affected by the ocean turbulence, our proposed mechanism eliminating the dependence on high-order OAM beam is also to a certain extent robust against the complex factors in underwater environment, which is crucial for its practical implementation in long-distance underwater communication. In our scheme, we have implemented four channels in a receiving area of 12*λ*_0_ × 5*λ*_0_, and the number of channels achieved in per unit area (normalized by ${\lambda }_{0}^{2}$) is about one order of magnitude higher than most OAM-based communication systems, which suggests a high spatial information density of our proposed mechanism. The spatial information density can be further improved by decreasing the distance between subunits (see Supplementary Note 10) and increasing the number of OAM modes in each transmission path. It is also noteworthy that the effective transmission distance of our proposed system can be further improved with no need of increasing the transmitter aperture by imposing low-diffraction vortex beam such as Bessel beam^40^ (see Supplementary Note 11).

## Discussion

In summary, we have proposed and experimentally demonstrated a mechanism using multipath twisting of acoustic wave to realize real-time free-space communication with high spatial information density in a passive postprocessing-free and sensor-scanning-free paradigm. The metamaterial, employed as the passive and efficient demultiplexing component for data decoding, not only has good performance in resisting inter-channel crosstalk but also can be compatible with conventional MIMO signal processing to further improve the accuracy of data transmission. The effectiveness of our proposed mechanism is demonstrated via real-time transmission of a complicated binary image. Despite the low number of transmission paths and short communication distance we choose to demonstrate here for simplifying the experimental fabrication and measurement, it is obvious that our scheme is general and can be straightforwardly applied to the design and implementation of large-scaled communication systems with a larger number of transmission paths and OAM modes. Moreover, our mechanism can apply to demultiplex the vortex beam of oblique incidence by compensating an additional deflection phase on the demultiplexer according to the generalized Snell’s law, which is crucial for underwater acoustic communication with multipath effect. Note also that the communication capacity and spectral efficiency can be further improved by using high-order shift keying to encode data. In addition to the extendability to break the limited bandwidth of underwater communication and ocean exploration, we also anticipate our proposed strategy to have far-reaching implications in diverse fields and to give important inspiration for relevant researches for other classical waves, such as integrated light communication devices with significant application potential to all-optical on-chip applications.

## Methods

### Numerical simulation

Throughout the paper, the simulations are conducted with a finite element method based on COMSOL MULTIPHYSICS software. The background medium is air, for which the mass density and sound speed are 1.21 kg/m^3^ and 343 m/s, respectively. The solid material for building the hybrid structure is chosen as UV resin whose mass density and sound speed are 1400 kg/m^3^ and 2000 m/s, respectively.

### Experimental configuration

The experiment is performed in an anechoic chamber without interference from other obstacles. The samples of two metamaterial-based demultiplexers are fabricated via 3D printing technology. Ten loudspeakers (HiVi, model B2S) are circularly-arranged on an acrylic glass plate and a single-chip microcomputer Arduino Mega 2560 is used to control the amplitude and phase of the drive signal of each loudspeaker. Four microphones (Brüel & Kæjr type-4961) are placed at the focal points to detect the signals. The real-time signals are recorded with Brüel & Kæjr PULSE 3160-A-042 4-channel analyzer.

## Supplementary information

Supplementary Information

## Data Availability

The data that supports the findings of this study are available from the corresponding author upon reasonable request.

## Code availability

The code that supports the findings of this study are available from the corresponding author upon reasonable request.
