# Supplementary material for: Metamaterial-based real-time communication with high information density by multipath twisting of acoustic wave
Source: Nat Commun. 2022 Sep 2;13:5171. doi: 10.1038/s41467-022-32778-z (PMC9440107; doi:10.1038/s41467-022-32778-z)
Supplement: Supplementary file 1 — Supplementary Information [file 41467_2022_32778_MOESM1_ESM.pdf]

Supplementary Information for

Metamaterial-based Real-time Communication with High Information Density by Multipath Twisting of Acoustic Wave

Kai Wu[*], Jing-Jing Liu[*†], Yu-Jiang Ding, Wei Wang, Bin Liang[†] and Jian-Chun Cheng[†]

Key Laboratory of Modern Acoustics, MOE, Institute of Acoustics, Department of Physics, Collaborative Innovation Center of Advanced Microstructures, Nanjing University, Nanjing 210093, *P. R. China*

[*]These two authors contributed equally to this work.

[†]Correspondence and requests for materials should be addressed to J.L. (email: liujingjing@nju.edu.cn), B.L. (email: liangbin@nju.edu.cn) or J.C. (email: jccheng@nju.edu.cn).

Supplementary Note 1. The compatibility of our proposed mechanism with multi-input multi-output (MIMO) equalization techniques.

In our multi-path system, N transmission paths are established between N pairs of transmitting and receiving units, referred to as T_i and R_i respectively with $i = 1, 2, \ldots, N$. Each transmitting unit emits a synthesized acoustic beam formed by coaxially overlapping vortices with different orders l_m with $m = 1, 2, \ldots, M$, resulting in a total number of NM orbital angular momentum (OAM) data channels. Because of divergency along the propagation distance, the vortex beams from different transmitting units may spatially overlap at the receiver. The energy collected by each receiving unit from the neighboring links manifests as channel crosstalk (or interference). The total crosstalk of each OAM channel can be estimated from the channel transfer matrix between NM OAM channels and the transfer matrix H can be written as

$$\mathrm{H} = \begin{bmatrix} h_{11} & h_{12} & \cdots & h_{1N} \\ h_{21} & h_{22} & \cdots & h_{2N} \\ \vdots & \vdots & \ddots & \vdots \\ h_{N1} & h_{N2} & \cdots & h_{NN} \end{bmatrix}_{NM\times NM} \quad (1)$$

where h_{ij} is an $M\times M$ matrix depicting the transfer function between OAM channels from unit T_j to R_i. In MIMO communication system, the optimal MIMO scheme can be obtained through the singular value decomposition of the transfer matrix between different channels, which can reduce the interference among channels and recover the transmitted data streams. But the computation complexity of the transfer matrix has to increase with the number of transmitting/receiving units. Ideally, in our proposed system, h_{ii} is a diagonal matrix benefiting from the orthogonality among the M transmitted coaxial OAM channels, meaning that H has N diagonal submatrices along its main diagonal line, which greatly simplifies the transfer matrix and results in a lower computation

complexity in the MIMO equalization technology. In our proposed mechanism, a metamaterial layer as a passive and efficient demultiplexer simultaneously removes the OAMs of all multiplexed vortex beams and spatially separate them into prescribed locations. Thanks to the robustness to the undesired crosstalk, our mechanism allows the real-time and high-accuracy decoding of transmitted data at the receiving end with no need of MIMO postprocessing or sensor scanning, such that the exemption from the time-consuming cost and computational complexity can be realized. Meanwhile, our strategy still maintains the compatibility with the optimal MIMO scheme to further equalize the crosstalk between different channels and has the advantage to reduce the computational complexity by simplifying the transfer matrix as ensured by the orthogonality among the vortex beams with different OAM modes.

Supplementary Note 2. Generation of multiplexed acoustic vortex beams with active loudspeaker array.

In our experiment we use 10 circularly-arranged loudspeakers (HiVi, model B2S) to assemble an active emitter unit as shown in Supplementary Fig. 1a. The radius of the ring array is 1.5 wavelengths. The relative phases of speakers used to emit acoustic vortex beam with OAM modes l are $\varphi = l\theta$, where θ is the azimuthal angle between the speaker and reference. The multiplexing of vortex beams with different OAM modes is accomplished by inputting the signals required to generate these modes in the single-chip microcomputer 1 (SCM1), which are then sent to the speakers to emit the multiplexed vortex beam. In our experiment, we use the relay to control the signals of SCM1 to be “on” or “off” to encode transmission data onto the amplitude of the vortex beams in 2ASK format. The relay is controlled by single-chip microcomputer 2 (SCM2) with transmission data being the input signal of SCM2. Here, the even-

numbered speakers are used to generate the vortex beam with OAM mode l = +1 and the odd-numbered speakers are used to generate the vortex beam with OAM mode l = -1, which are staggered along the azimuthal direction. Supplementary Figure 1b shows the relative phase of input signals of ten speakers, wherein all speakers emit with identical amplitude.

a

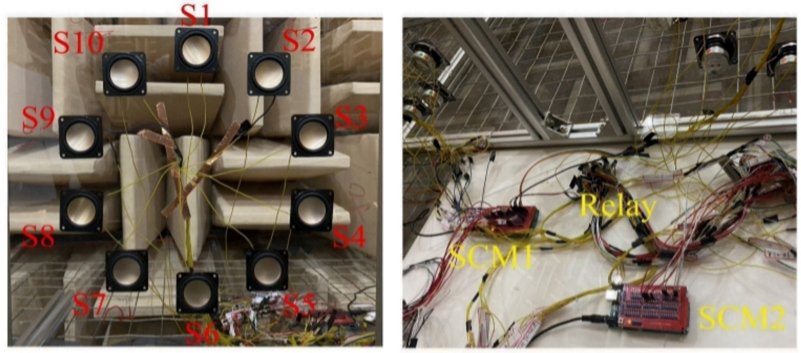

b

	S1	S2	S3	S4	S5	S6	S7	S8	S9	S10
φ/rad	0	$\pi/5$	$8\pi/5$	$3\pi/5$	$6\pi/5$	π	$4\pi/5$	$7\pi/5$	$2\pi/5$	$9\pi/5$

Supplementary Figure 1 | Active transducer array used in our experiment. a The SCM1 on the left drives ten speakers marked by S1-S10 on the acrylic glass frame. Ten relays controlled by SCM2 are used to control ten input signals from SCM1 to be "on" or "off" independently and respectively. **b** The relative phase of input signals of ten speakers.

Supplementary Note 3. Physical mechanism of the acoustic metamaterial-based demultiplexer.

The metamaterial-based demultiplexer possesses the ability of demultiplexing and focusing vortex beams with different OAM modes into the prescribed focal points. The mechanism of the demultiplexer is based on manipulating the phase function distributed on the metamaterial to perform simultaneous demultiplexing and focusing of multiplexed vortex beams. In order to convert the l-th OAM beam to focusing beam, one can multiple an inverse helical phase $e^{-il\theta}$ by focusing phase based on the

superposition principle. Due to the intrinsic orthogonality of OAM, the synthesized phase profile can be generated by adding inverse helical phases and focusing phases of different OAM modes together. In our experiment, we deign the metamaterial to demultiplex the multiplexed vortex beams with OAM modes $l_1 = +1$ and $l_2 = -1$ and focus them into point F_1 (0, 1.5λ_0, 2λ_0) for OAM mode $l_1 = +1$ and point F_2 (0, -1.5λ_0, 2λ_0) for OAM mode $l_2 = -1$ (λ_0 is the wavelength of sound wave with frequency of 3430 Hz). Then the phase function distributed on the metamaterial-based demultiplexer can be expressed as

$$\Phi = \arg(\exp(i(-l_1)\theta + ikr_1) + \exp(i(-l_2)\theta + ikr_2)) \quad (2)$$

where k is the wavevector in free space and r_1, r_2 are the distances between the demultiplexer and the focal points F_1 and F_2, respectively. The detailed process of generated profile is illustrated in Supplementary Figure 2. Considering the finite size of each building block of demultiplexer, the continuous phase distribution in the metamaterial is discretized with a discrete degree of 0.25λ_0, since this spatial resolution is good enough for the metamaterial to enable effective beam untwisting and focusing manipulation. Besides, four equally spaced phases (0, π/2, π, 3π/2) are also applied to replace the continuous phase profile.

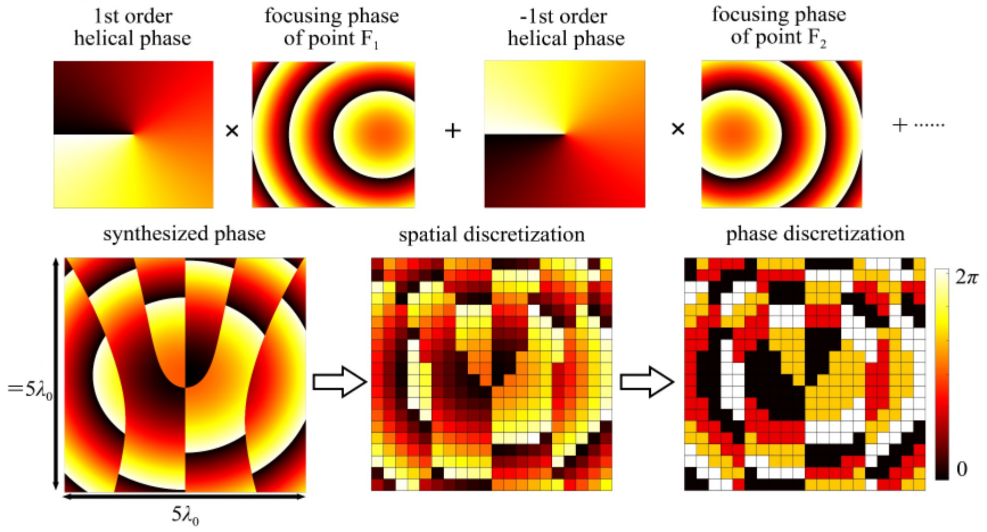

Supplementary Figure 2 | Schematic diagram of derivation and generation of phase distribution on metamaterial-based demultiplexer for demultiplexing and focusing two-OAM-beam.

Supplementary Note 4. The design of building blocks of metamaterial.

Supplementary Figure 3a illustrates the configuration of the designed metamaterial unit cell that is composed of one row of acoustic Helmholtz resonators mounted on a straight channel. Each unit consists of four Helmholtz cavities and a straight pipe and the phase characteristic of metamaterial is induced by the acoustic hybrid resonance between the Helmholtz cavities and straight pipe. The free manipulation of phase can be achieved by adjusting the single geometrical parameter of the metamaterial unit cell. Here we fix the thickness d of the wall to be $0.015\lambda_0$ and the thickness D of the resonator to be $0.5\lambda_0$. Then we choose to modulate the height h of straight pipes to effectively realize the manipulation. We simulate the phase shift and transmission coefficient of the metamaterial unit cell as a function of the height of straight pipes in Fig. 1c. We use four values of h marked by the four dots in Fig. 1c in the main text to achieve four steps of an equally spaced phase shift for practical realization. The simulated field distributions through these four elements are illustrated in Supplementary Fig. 3b. The simulated acoustic pressure fields show the equally-spaced phase shift from 0 to 2π as well as a high transmission over 90%.

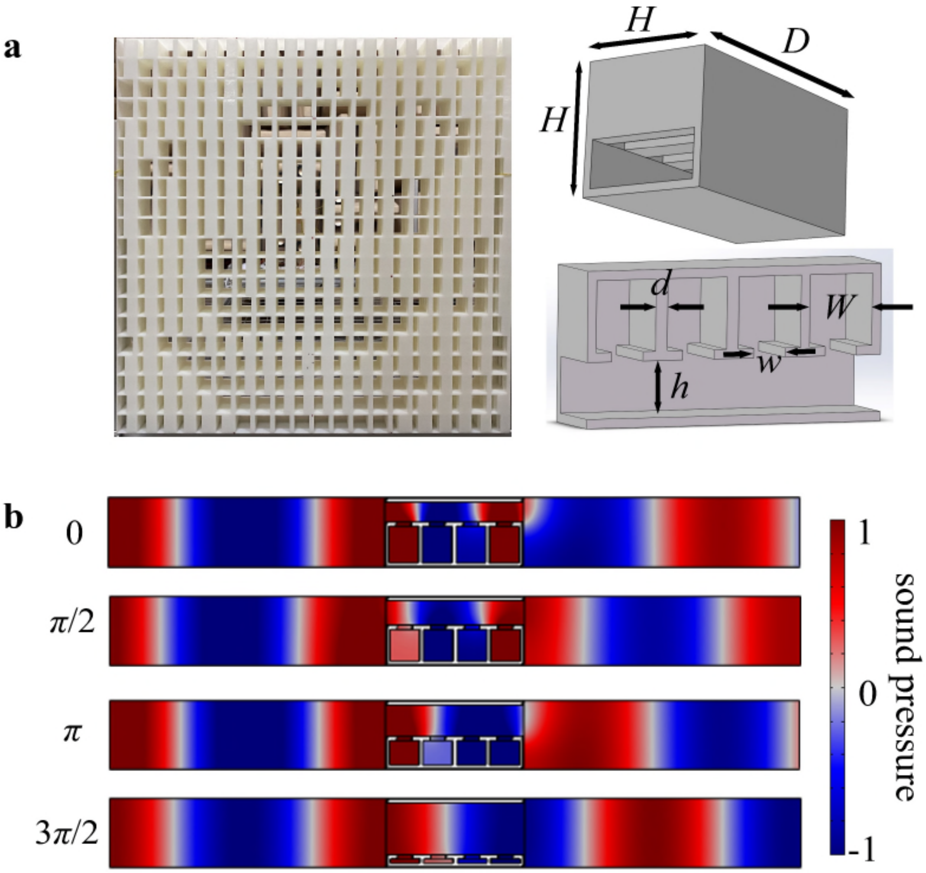

Supplementary Figure 3 | a Photograph of the fabricated sample of designed metamaterial-based demultiplexer consisting of 20 × 20 hybrid resonators. Inset: illustrative diagram of acoustic hybrid resonator whose structural parameters are set as $H = 0.25\lambda_0$, $D = 0.5\lambda_0$ and $w = W/2$. **b** Simulated sound transmission through individual elements for an equally increased phase shift with a step of $\pi/2$, corresponding to the four dots in Fig. 1c with h = 7.3 mm, 9 mm, 11.45 mm, and 19 mm. Plotted snapshot of the sound field is referenced to the amplitude of incident plane waves.

Supplementary Note 5. The spreading and overlapping of vortex beams during propagation.

In our proposed system, the radial size of transduce array is $1.5\lambda_0$ and this limited size will cause inevitably the severe diffraction effect during propagation. To illustrate the influence of diffraction over longer transfer distances, we first give the *x*-*y* plane sound pressure distributions at two

transmission distances of $12\lambda_0$ and $20\lambda_0$ when one channel, two non-coaxial channels and four channels are opened respectively, as shown in Supplementary Fig. 4. It can be clearly observed that after propagating $20\lambda_0$, the mainlobe of the single-mode vortex beam has exceeded the demultiplexer (transverse size is 0.5 m × 0.5 m) and the non-coaxial vortex beams have overlapped seriously in space, but the long-distance communication system still has good performance in terms of information density and BER, which has been experimentally demonstrated in the transmission of binary image of Nanjing University logo. Then, in Supplementary Fig. 5, we also provide the *x*-*z* plane sound pressure distributions for three particular cases with one channel, two non-coaxial channels and four channels opened respectively, to exhibit the diffraction and overlapping of acoustic waves during propagation.

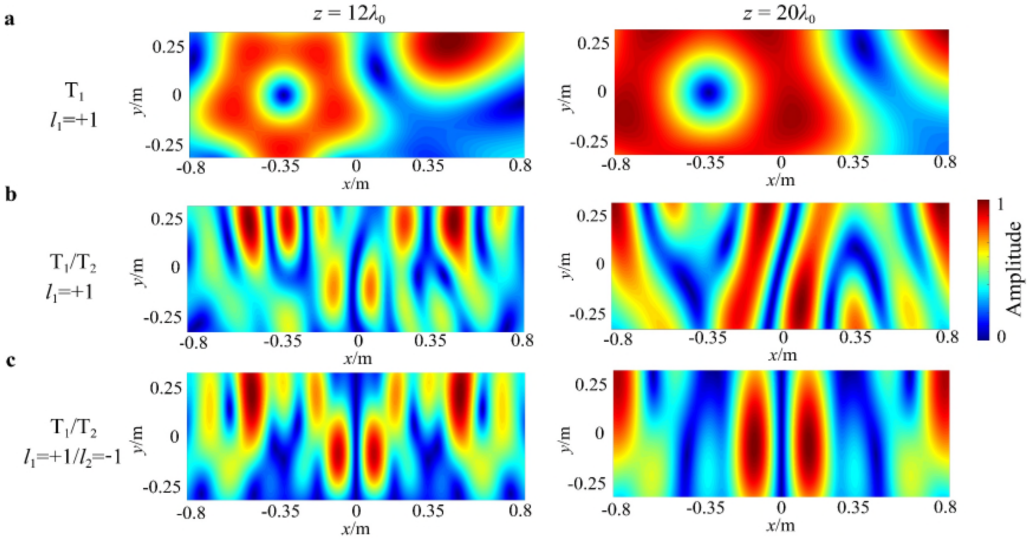

Supplementary Figure 4 | The simulated *x*-*y* plane sound pressure distributions in our proposed communication system with $12\lambda_0$ and $20\lambda_0$ transmission distances respectively, when **a** one channel, **b** two non-coaxial channels and **c** all four channels are opened.

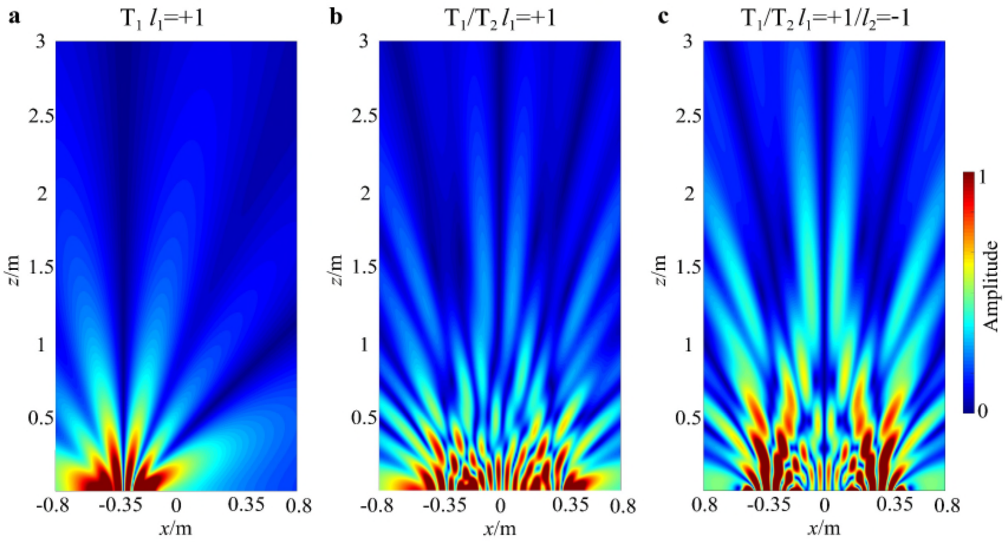

Supplementary Figure 5 | The calculated *x*-*z* plane sound pressure distributions for three cases with **a** one channel, **b** two non-coaxial channels and **c** all four channels opened respectively.

Supplementary Note 6. Generation of 686 bit/s 2ASK signal at 3430 Hz carrier frequency.

The input multiplexed signal is in pulse modulation, with the central frequency f_0 = 3430 Hz (period of T_0 and wavelength of 10 cm) and pulse period of 20 T_0, wherein each pulse cycle contains one-bit data. In order to generate 2ASK signal, we use relays to control inputting signals of speakers to be "on" or "off" independently and respectively to encode transmission data onto the amplitude of the vortex beams. The relay is controlled by SCM2 with transmission data being the input signal of SCM1. In our experiment, we encode four data streams (Fig. 3 in the main text) into the amplitude of vortex beams with OAM modes l_1 = +1 and l_2 = -1 of two transmitting units T_1 and T_2. The ten inputs for generating the multiplexed signal of T_1 are shown in Supplementary Fig. 6a, from which we can observe that the ten inputs have a relative phase difference of $\pi/5$ varying from 0 to $9\pi/5$. The input signals of S2, S4, S6, S8 and S10

are modulated by the first data stream (Fig. 3) in 2ASK format to generate the -1st-order vortex beam carrying information. Other inputs are used to generate the 1st-order vortex beam carrying the second data stream. Then, the Supplementary Figure 6b shows ten inputs of transmitting unit T_2 to generate the multiplexed signals of OAM $l_1 = +1$ and $l_2 = -1$, modulated by the third and the fourth data streams respectively.

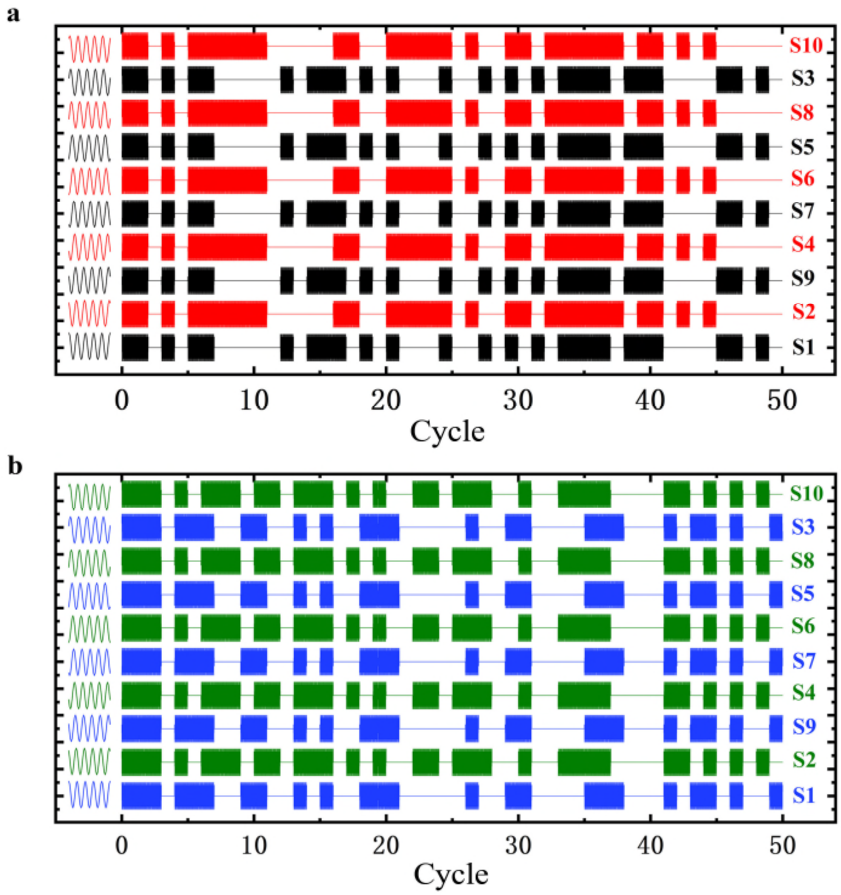

Supplementary Figure 6 | Ten inputs of transmitting unit T1 **a** and transmitting unit T2 **b** for generating the multiplexed signal modulated by four data streams (Fig. 3 in the main text) in 2ASK format respectively. The signals are in pulse modulation, with the central frequency f_0 (period T_0), pulse period $20T_0$. Left inset: An enlarged view of the input signal for each speaker.

Supplementary Note 7. Extracting the data stream from time-domain signal with cross-correlation method.

The time-domain signals received by each microphone located at focal point directly reflect the amplified signals transmitted over the corresponding channel without loss of encoded information. Here, we perform the cross-correlation between time-domain signals and an ideal sinusoidal signal with frequency of 3430 Hz to extract the transmission data from time-domain signals. The results of correlation spectra are shown in Supplementary Fig. 7, where the length of the sinusoidal signal is equal to the length of one pulse period of the received signal. We use a threshold of 0.5 to determine that the transmitted information is “1” or “0”.

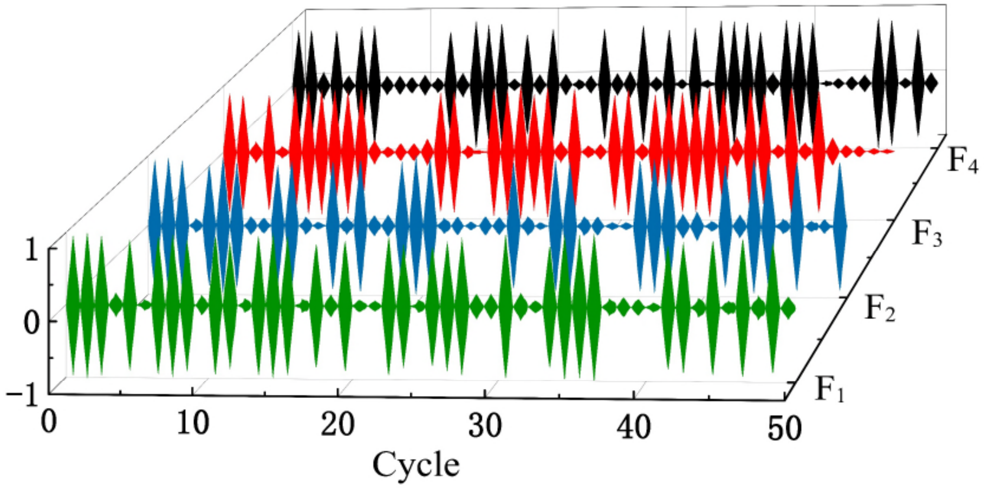

Supplementary Figure 7 | The waveforms of correlation function between the time-domain signals received at four focal points and an ideal sinusoidal signal with the frequency of 3430 Hz.

Supplementary Note 8. The realization of higher communication speed of our design.

There are three main reasons leading to the relatively low communication speed in our design: (1) Low working frequency. To avoid high attenuation of airborne sound at ultrasound regime and difficulty in sample fabrication, in the experiment demonstrated in the main text, we choose the working frequency as 3.43 kHz, orders of magnitude lower than

the frequency commonly used for underwater communications. (2) The number of channels. For simplicity while without losing generality, we built a four-channel communication system including two non-coaxial transmission paths with each transmitting two co-axially overlapped vortex beams. Indeed, more channels can be established by increasing the number of non-coaxial transmission paths and the number of OAM modes in each path. For example, by redesigning the phase mask with no need of changing the link number the proposed mechanism can realize effective mode separation and decoding for more OAM modes, as evidence by the OAM demultiplexing results for an upgraded two-path four-mode (N = 2, M = 4) system shown in Supplementary Fig. 8a. (3) Low-order modulation technology. In our proposed system, the vortex beam is modulated to carry 2-ary amplitude-shift keying (2ASK) signals for simplifying experimental measurements, which contains 1-bit data in each pulse cycle. The communication speed can be easily multiplied once we apply high-order modulation such as 4ASK into the designed system. To verify this, we also numerically calculate the sound pressure amplitudes at focal points when four-mode vortex beam carrying 4ASK signals is incident on the redesigned demultiplexer and the results in Supplementary Fig. 8b show the improved capacity of coding 2-bit data per ASK symbol. As a result, if we increase the working frequency to 23 kHz and choose 4ASK modulation format for eight channels, the communication speed can reach 46 kbit/s, much higher than most of the existing acoustic MIMO communication systems. In other words, when we keep other parameters the same as that of traditional MIMO systems, more available channels in our system can guarantee higher communication speed. Take one MIMO communication system with two inputs and eight outputs, that is, two independent channels as instance [see Ref. 33 in the main text], they choose the working frequency to be 23 kHz and use quadrature phase shift keying

(QPSK) to encode data, and finally the achieved data rate is 11.15 kbit/s, about one quarter of our communication speed. Therefore, our mechanism opens an avenue to metamaterial-based high-capacity especially high spatial information density communication paradigm, where the high capacity is realized by a larger number of achievable OAM channels and the compatibility of proposed mechanism with existing modulation techniques.

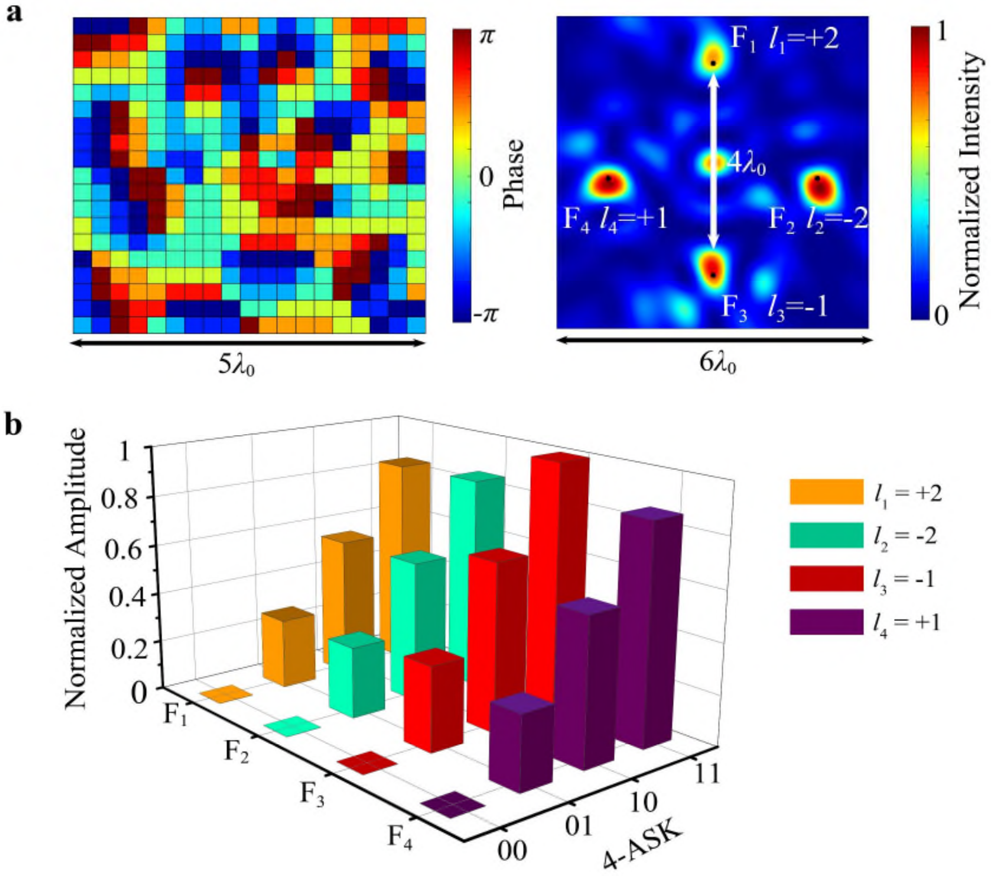

Supplementary Figure 8 | a Discrete phase profile and demultiplexing result of redesigned demultiplexer for four-mode vortex beam, which ensures the performance of eight-channel communication system. **b** The normalized amplitude at four focal points when four-mode vortex beam carrying 4ASK signals is incident on the redesigned demultiplexer, which demonstrates the effectiveness of applying high-order amplitude shift keying in eight-channel communication system.

Supplementary Note 9. The realization of accurate underwater acoustic communication over a km-scale distance with the optimal arrays dimensions.

The real-time free-space communication system using a transmitting array with the radial size of 3 wavelengths in the main text has realized 20-wavelength transmission distance and high communication speed, thanks to the proposed mechanism that uses multipath twisting of acoustic wave to avoid high-order OAM beams for attaining an equivalent number of communication channels. Here, we give further investigation on the potential of our mechanism to realize high-speed communication at a distance much longer than the current optical technology of underwater communication without enlarging unlimitedly the arrays for sending and receiving signals. We quantitatively evaluate the relationship between the distance of effective communication and the size of transmitting/receiving array and demonstrate in what follows how to achieve the optimal communication distance with high accuracy by using transmitting/receiving arrays with applicable size via numerical simulations (instead of experiments due to the limited experimental space).

We introduce a parameter *R* defined as the ratio between the focus sound intensity for the one opened channel and the average focus intensity for the rest closed channels to evaluate the decoding accuracy of system. Then we calculate the numerical results of *R* versus the transmission distance under different array dimensions and plot the typical results in Supplementary Fig. 9a. The value of sound intensity ratio can reflect the decoding performance of the receiving end and the accuracy of data decoding will decrease with the decrease of intensity ratio. In our simulation, the multiplexed OAM modes are still chosen as $l = +1$ and $l = -1$ as in the main text and the working frequency is set to be 18 kHz which is a common frequency for underwater communication (corresponding to a wavelength

λ of 8.3 cm in water). From Supplementary Fig. 9a, it can be clearly observed that the intensity ratio shows a downward trend with the increase of the transmission distance and the decrease of array dimension, which suggests a longer transmission distance can be achieved by using a larger size transmitting array. We choose the intensity ratio equal to 1 as the criterion of the effective transmission distance which means the sound intensities measured at the opened channel and closed channels are nearly identical as the BER of data decoding will increase rapidly when $R<1$. Then we also plot in Supplementary Fig. 9b the communication distance versus the transverse dimension (*TD*) of array when the intensity ratio is equal to 1. We observe that the effective transmission distance does not increase linearly with the transverse dimension of array, implying the possibility to greatly increase the transmission distance with optimally-designed array dimensions.

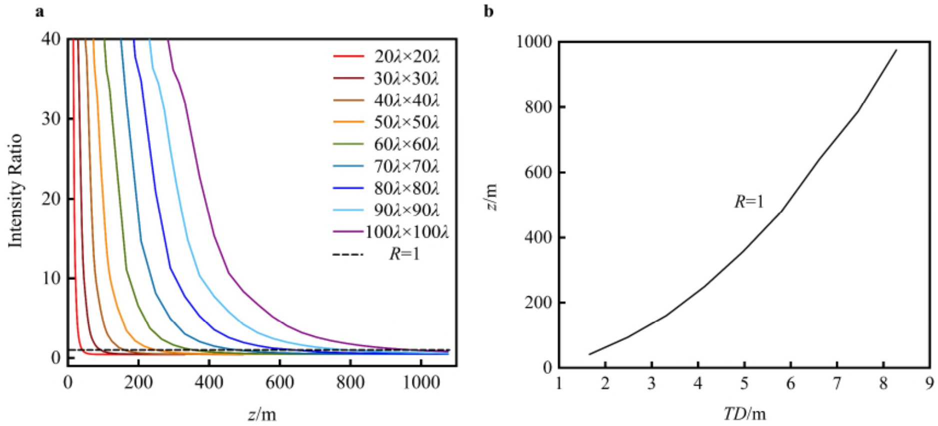

Supplementary Figure 9 | a The intensity ratio as a function of the transmission distance for different array dimensions. **b** The transmission distance versus the transverse size of array when R = 1.

As a verification, we demonstrate via numerical simulations the effectiveness of our mechanism to realize high-speed acoustic communication at a transmission distance much longer than achievable with existing optical communication systems based on appropriately-sized

arrays. The distance between the inner edges of transmitting units T_1 and T_2 is still 2λ. The multiplexed vortex beams emitted from T_1 and T_2 with transverse sizes of $95\lambda \times 95\lambda$ (7.86 m × 7.86 m) are propagating toward the receiving units R_1 and R_2 over a transmission distance of 11000λ (910.5 m). This source array can be carried by submarines, which makes it possible to practically implement our mechanism in underwater acoustic communication. And this transmission distance exceeds the longest underwater optical communication distance realized by using blue-green laser with small absorption and scattering coefficient in clear oceanic medium. In turbid ocean water, furthermore, the contrast between the performance of our designed system and optical communication system will be further increased due to the severe water absorption and particle scattering of light wave. At the receiving end, we redesign the metamaterial layer for this long-distance communication system, and the two-path synthesized vortex beams are demultiplexed and focused into the expected detection points, i.e., F_1 (-41λ, 30λ, 30λ) for OAM mode l = +1 from T_1, F_2 (-41λ, -30λ, 30λ) for OAM mode l = -1 from T_1, F_3 (41λ, 30λ, 30λ) for OAM mode l = +1 from T_2 and F_4 (41λ, -30λ, 30λ) for OAM mode l = -1 from T_2. To prove the effectiveness of this communication system, we calculate the sound intensity at four focal points under all encoding circumstances of two-path two-mode multiplexed system and plot the histograms of normalized sound intensity in Supplementary Figs. 10a-c. Supplementary Figure 10a illustrates the intensity response at four points for the case of only one opened channel. The results show that the sound intensity difference between channel "on" and "off" exceeds 16 dB, which guarantees the low BER in the practical implementation of the multi-path OAM-based communication. Supplementary Figures 10b and 10c show the demultiplexing results of multi-channel system, proving that the sound intensity also has a high contrast between the channel "on" and "off". For

clearer illustration, we also plot in Supplementary Fig. 11 the simulated sound intensity distributions on the output plane when turning on a single channel, two channels, three channels and four channels respectively, which manifest the ability of demultiplexers to separate and focus the vortex beams with different OAM modes into predesigned focal points. Therefore, by optimally choosing the size of source array, we increase the transmission distance by 220 times and the ratio of transmission distance to the aperture size by more than one order of magnitude, which also proves the nonlinear relationship between transmission distance and the aperture size in Supplementary Fig. 9b.

The good BER performance over long-distance communication we declare is ensured by the high spatial selectivity, high robustness and decoding accuracy of our mechanism, which has been partially proved by the accurate demultiplexing results for all channels. Here for better verification, we also calculate the crosstalk of each OAM channel in the long-distance communication system to characterize the channel quality, which also affects the BER performance of the system, and plot the results in Supplementary Table 1. From the results we can observe that the crosstalk of four OAM channels is below -9 dB, with no significant difference as compared with the previous short-range system with crosstalk of about -10 dB (Table 1 in the main text), which suggests that our mechanism has good channel quality in long-distance communication. Besides, our mechanism has a certain tolerance to the complex factors in underwater environment such as volume scattering and turbulence, and the BER performance is expected to be further enhanced thanks to the compatibility of our mechanism with the existing MIMO equalization algorithms or machine learning-based techniques at the receiving end. Based on these, we conclude that the BER performance of our communication system, which is mainly determined by the channel quality,

SNR and transmission medium, should keep a high level in long-distance communication despite not experimentally demonstrated due to the limited experimental space.

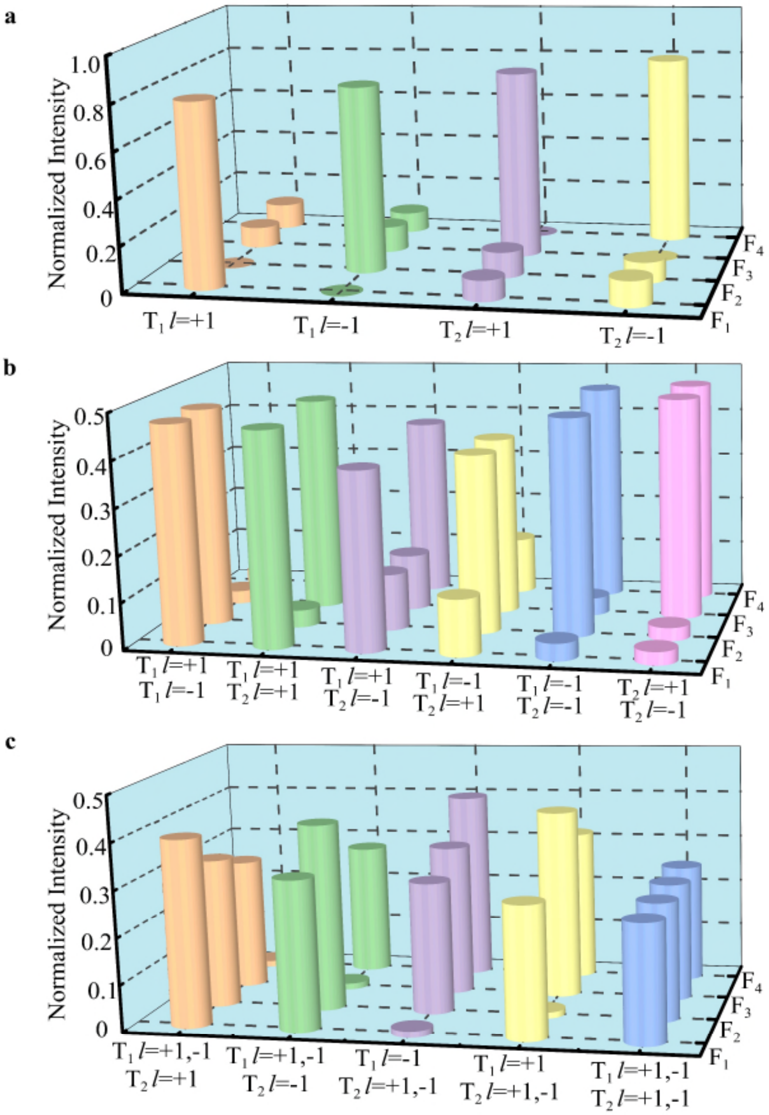

Supplementary Figure 10 | Histograms of normalized sound intensity at four focal points when **a** only one channel is opened, **b** two channels are opened and **c** three channels and all four channels are opened.

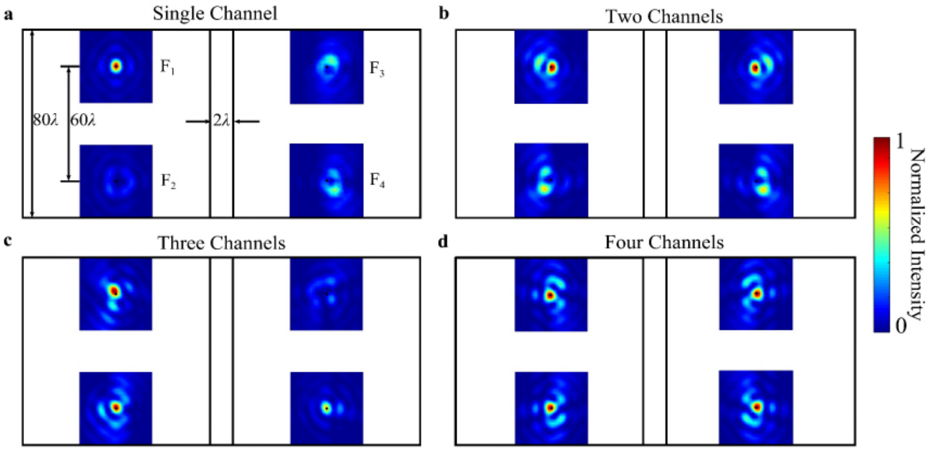

Supplementary Figure 11 | The simulated sound intensity distributions on the output plane when turning on **a** single channel, **b** two channels, **c** three channels and **d** four channels respectively.

OAM channel	R_1 l_1 = +1	R_1 l_2 = -1	R_2 l_1 = +1	R_2 l_2 = -1
Crosstalk (dB)	-9.49	-9.03	-9.8	-9.29

Supplementary Table 1 | The crosstalk of OAM channels in the long-distance underwater communication system.

Supplementary Note 10. The influence of the distance between transmitting/receiving units on the communication performance.

In our proposed system, we set the spacing distance of two transmitting (receiving) units to be $2\lambda_0$ to facilitate the proof-of-concept experiment, but we can reduce the spacing distance to make the aperture size smaller while keeping the communication performance, which is ensured by the robustness of our mechanism to crosstalk. Typical results are shown in Supplementary Figs. 12a and b, which illustrates the simulated *x*-*z* plane sound pressure distributions and the demultiplexing results on the output plane for three particular distances between subunits: $D = \lambda_0$, $0.5\lambda_0$ and 0

respectively. Obviously, the spatial overlapping of sound waves becomes more serious with the decrease of spacing distance, resulting in more crosstalk between channels. However, from Supplementary Fig. 12b we can observe that the reduction of spacing distance has little effect on the demultiplexing results even when the spacing distance approaches 0. The robustness of our system to crosstalk also offers the possibility of downscaling the transmitting and receiving terminals, enabling a compact acoustic communication system.

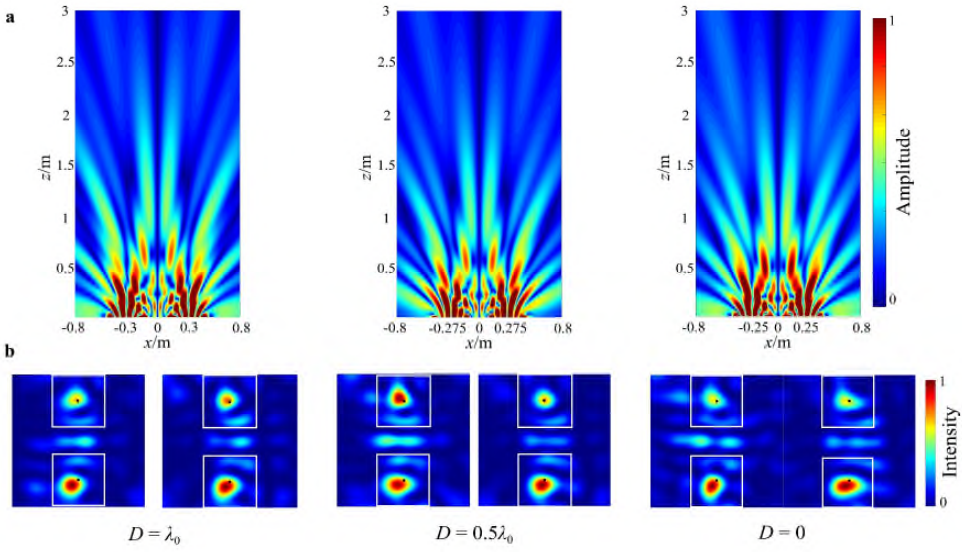

Supplementary Figure 12 | The system performance for three particular distances between subunits: *D* =λ_0, 0.5λ_0 and 0. a The calculated *x*-*z* plane pressure distributions of our proposed system with four channels opened simultaneously. **b** The sound intensity distributions on the output plane when the transmission distance is 12λ_0 and four channels are opened simultaneously.

Supplementary Note 11. The performance of our proposed mechanism using low-diffraction vortex beam as carrier.

Due to the size limitation of our anechoic chamber, it is not feasible to experimentally demonstrate the effectiveness of our communication system at a longer transmission distance. Instead, we performed a series of numerical simulations to verify the performance of our system at a longer

transmission distance (e.g., $50\lambda_0$). From the typical results in Supplementary Fig. 13a showing the simulated spatial distribution of sound intensity on the output plane with four OAM channels opened simultaneously, it is observed that thanks to the strong spatial selectivity of our designed monolayered demultiplexer, the sound intensity at the predesigned point is significantly enhanced as expected. This fully manifests that the channel crosstalk caused by the diffraction effect has negligible influence on the detection accuracy at focal points, which ensures the performance of the communication system even when the communication distance reaches $50\lambda_0$. It is noteworthy that upper limit of transmission distance can be further improved without increasing the transmitter aperture by imposing low-diffraction vortex beam. Here we calculate the relationship curves of mainlobe width of first-order Bessel beam as well as current vortex beam versus the transmission distance, which is a common parameter to evaluate the diffraction of vortex beam. The typical results in Supplementary Fig. 13b show that the mainlobe of Bessel beam expands more slowly than current vortex beam with the propagation distance increasing, which implies the possibility of further increasing the communication distance by applying Bessel beam.

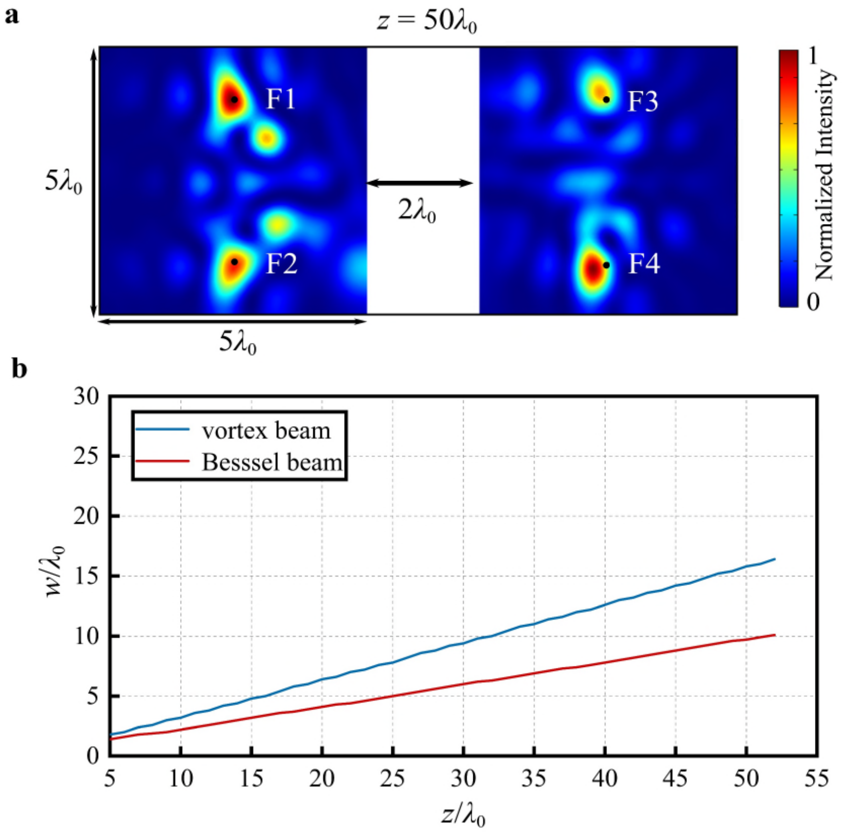

Supplementary Figure 13 | a The simulated sound intensity distributions on the output plane with four channels opened simultaneously and a transmission distance of $50\lambda_0$. **b** The mainlobe width of Bessel beam and current vortex beam versus transmission distance.
